# 251 Development of the Physical Activity Messaging Guide to Enhance Messaging Practice across HEPA Nations

**DOI:** 10.1093/eurpub/ckae114.275

**Published:** 2024-09-26

**Authors:** Chloë Williamson

**Affiliations:** The Physical Activity for Health Research Centre, University of Edinburgh, Scotlandchloe.williamson@ed.ac.uk

**Keywords:** Exercise, communication, toolkit, resource

## Abstract

**Purpose:**

Physical activity (PA) messaging can help change individual and social factors, and thus contribute to improving PA levels, but improving PA messaging practice is warranted. In 2021, the Physical Activity Messaging Framework was published following a robust modified Delphi study. One purpose of this framework is to aid creation of new PA messages. However, a need to enhance dissemination and uptake of this framework in day-to-day practice was identified. Therefore, this project aimed to translate the principles of the framework to develop a practitioner-friendly messaging guide.

**Methods:**

The framework was adapted and combined with existing evidence to create a step-by-step guide; the Physical Activity Messaging Guide (PAMG). Following this, formal feedback on PAMG was sought through online and in person meetings with members of HEPA/WHO Europe. This feedback was acted on to develop a further draft, which was shared more widely via an online, open-questioned survey (Qualtrics) to gather broader feedback.

**Results:**

At time of writing, feedback has been gathered from 39 individuals via online survey. Responses were gathered from the UK (including Scotland, Northern Ireland, and England), the United States, Ireland, Germany, Romania, Spain, Portugal, Canada, Australia, and Finland, and came from a mixture of academics, exercise professionals, healthcare professionals, practitioners, and other (e.g., education) professions. Feedback was overall positive, with constructive recommendations for further refinement. Amendments are underway based on feedback to develop a final version of the PAMG, which will be presented at the HEPA 2024 Conference in Dublin, Ireland. Key next steps include translation of the guide into different languages to further enhance uptake.

**Conclusions:**

Involvement in the Early Career Professional Development Programme has enabled a collaborative approach to develop and refine the PAMG. The PAMG will, as a result, be a more useful tool to various users across countries and contexts. If the PAMG is applied consistently in messaging practice, developed PA messages will be more evidence-based and target-audience focused, ultimately contributing to improving population levels of PA.

**Funding Source:**

N/A. This work was completed as part of CW’s appointment to the HEPA/WHO Europe Early Career Professional Development Programme 2024.

